# Evidence of shared bovine viral diarrhea infections between red deer and extensively raised cattle in south-central Spain

**DOI:** 10.1186/s12917-015-0630-3

**Published:** 2016-01-14

**Authors:** Víctor Rodríguez-Prieto, Deborah Kukielka, Belén Rivera-Arroyo, Beatriz Martínez-López, Ana Isabel de las Heras, José Manuel Sánchez-Vizcaíno, Joaquín Vicente

**Affiliations:** VISAVET, Veterinary School, Complutense University of Madrid, Puerta de Hierro s/n, 28040, Madrid, Spain; SaBio (Sanidad y Biotecnología) research group, National Wildlife Research Institute IREC (CSIC - Universidad de Castilla-La Mancha), Ronda de Toledo s/n, 13071, Ciudad Real, Spain

**Keywords:** Bovine viral diarrhea, Pestivirus, Taqman RT-PCR, ELISA, GLMM, Wild ungulates, Wildlife reservoirs, Spain

## Abstract

**Background:**

Bovine viral diarrhea virus (BVDV) is a pestivirus that affects cattle production worldwide and that can infect other ungulates such as cervids and even wild boar (*Sus scrofa*). It is believed that domestic livestock can become infected through contact with wild animals, though it is known that infection can spread among wild animals in the absence of contact with livestock. Little is known about the sharing of BVDV infection between wild and domestic animals in the same habitat, which is important for designing eradication campaigns and preventing outbreaks, especially on hunting estates with high animal densities.

**Results:**

We assessed the sharing of BVDV infections among hunted red deer, wild boar and cattle in south-central Spain. Sampled red deer (*Cervus elaphus*; *n* = 267) and wild boar (*n* = 52) were located on 19 hunting estates, and cattle (*n* = 180) were located on 18 nearby farms. We used ELISA kits for the serological screening, Taqman RT-PCR assay for the virus determination, and subsequent phylogenetic analysis for 17 RT-PCR positive sample amplicons. Fifty-two red deer (19.5 %) and 82 cattle (45.6 %) samples tested positive by ELISA. A high apparent prevalence (22.47 %) was obtained for red deer, while only five cattle farms tested positive by RT-PCR. Conversely, no wild boar tested positive by both ELISA or RT-PCR. Eleven red deer (4.1 %) tested positive by both ELISA and RT-PCR; these animals may have been sampled during the last phase of viremia, or they may represent previously exposed individuals infected by a different BVDV strain. The amplicons shared 92.7–100 % identity and fell within the BVDV subgroup 1b, although nine of these (from four red deer and five cattle pools) formed a separate branch. This suggests that there might be a common BVDV infecting both cattle and red deer. Higher red deer abundance was significantly associated with greater risk that extensively raised cattle would test positive for BVDV by ELISA.

**Conclusions:**

Our findings suggest that BVDV is circulating between cattle and red deer populations in proximity, but further work is required to determine whether they share the same strain(s). These results suggest the potential of BVDV to serve as a surveillance marker in these shared habitats. High seroprevalence of BVDV in red deer from our study area suggests that although BVDV infection is common, animals usually survive the infection. Further research is needed to verify and investigate the role of red deer as a BVDV reservoir.

## Background

*Pestivirus*, a genus of RNA viruses belonging to the *Flaviviridae* family, comprises four major members: bovine viral diarrhea virus (BVDV) genotypes one and two, border disease virus (BDV) and classical swine fever virus (CSFV) [1]. Pestiviruses are characterized by affecting a wide variety of ungulates. Specifically, BVDV mainly affects cattle, causing a significant economic impact on livestock production. This disease is considered endemic in many European regions, reaching values up to 60–85 % seropositive cattle [2]. Many European countries have implemented eradication campaigns focused on removing persistently infected (PI) calves. Although PI animals usually make up only 0.5–2 % of cattle populations [2], this proportion is enough to perpetuate BVDV circulation over time, due to the high viral excretion occurring in these individuals. Recently, other modes of transmission have been proposed, such as contact between cattle and infected wild hosts [3].

Transmission of pathogens between wild and domestic hosts is an area of growing concern all over the world [4]. Many current research programs are trying to evaluate the role of wild hosts in the persistence and transmission of infections shared with domestic animals. As more information is gained, more species are designated as reservoirs of infectious diseases, widening classical host ranges. Pestiviruses, originally categorized based on their domestic host, have been shown to infect a wide range of hosts [5], suggesting interspecies transmission. In the case of BVD, serological surveys in free-ranging and captive populations have detected infection in more than 40 species [3]. This list includes a wide variety of ruminants, especially cervids, although other species such as wild boar (*Sus scrofa*) or rabbit (*Oryctolagus cuniculus*) have been proposed as carriers [6]. Thus, the interaction between domestic livestock and wild animals has been proposed as a source of BVDV infection [7, 8]. Interaction may be direct or indirect via common pasturing and shared watering areas [9]. Understanding this “sharing” of BVDV between wild and domestic animals living in proximity is key in order to design effective eradication strategies in domestic populations.

Complicating our understanding of whether and how such BVDV sharing occurs, several studies suggest that contact with livestock is not always required for BVDV infection of wild hosts. Frölich [10] found that BVDV seroprevalence rates in free-ranging deer were not associated with cattle density, suggesting that the virus can be maintained in deer populations without contact with cattle. Elazhary et al. [11] also observed high BVDV seroprevalence in free-ranging Canadian caribou (*Rangifer tarandus*) that showed no documented contact with domestic cattle for 25 years. These findings suggest that BVDV can be maintained in wild populations without transmission from cattle. BVDV spread can even be enhanced when animal densities are high, so any management measure that increases wild ruminant densities and/or aggregation may favor viral circulation within a population. Such management practices, often intended to increase hunting bags, include fencing, supplementation of feeding and watering and animal translocation [12, 13]. When not properly controlled, this human-derived management leads not only to wild species overabundance, but also to create an optimal epidemiological scenario for pathogen transmission.

In Europe, wild boar and deer constitute the major big game species. To satisfy recreational hunting demand, these species are frequently bred under more intensive conditions, but under sometimes inadequate health conditions [4]. In addition, hunting estates usually lie adjacent to farms with extensively raised livestock. In these agroforestry systems wild ungulates can easily interact with extensively raised livestock, leading to the transmission of pathogens from domestic to wild populations or vice versa.

Breeding of red deer (*Cervus elaphus*) for hunting in Europe raises the question of whether this species may serve as a significant BVDV reservoir that may help drive infections in cattle populations. Vertical BVDV transmission has been experimentally demonstrated from pregnant white-tailed deer (*Odocoileus virginianus*) to fawns, which became PI as a result [14–16]. The pregnant deer in these studies had become infected by direct inoculation [14], or through contact with PI calves [15] or PI fawns [16]. These studies suggest that red deer may play a key role in BVDV epidemiology, but further work is needed to determine the details of that role and examine whether infected red deer can transmit the virus horizontally into other animal populations sharing the same habitat. This would allow to identify areas at high risk and increase opportunities to control disease transmission.

The aims of the present study were to (i) detect BVDV circulating in extensively raised cattle and red deer cohabiting, (ii) examine the epidemiological role of wild ungulates and cattle living in proximity concerning BVD, and (iii) assess the potential for using BVDV as a health marker in habitats where wildlife (i.e. red deer and wild boar) co-exist with domestic cattle. The insights gained from this work may advance research on the interactions at the wildlife-cattle interface and aid in the design of husbandry and health policies for more cost-effectively disease control.

## Results

We assessed the sharing of BVDV infections among hunted red deer, wild boar and cattle in south-central Spain. Sampled red deer (*n* = 267) and wild boar (*n* = 52) were located on 19 hunting estates, and the cattle (*n* = 180) were located on 18 nearby farms. Anti-BVDV antibodies were detected in 52 deer (19.5 %; 95 % CI = 14.7–24.2) and 82 cattle (45.6 %; 95 % CI = 38.3–52.8), but not in any wild boar. Table 1 provides data on overall seroprevalence and apparent prevalence in red deer as a function of estate type (fenced or unfenced), hunting season, age, and sex.

**Table 1:** Diagnostic test results for BVDV detection in red deer

Variable	Category	Total samples	No. PCR-positive (%)	No. ELISA-positive (%)
Species	Red deer	267	60 (22.47)	52 (19.47)
Region	MT	154	40 (25.97)	27 (17.53)
	SM	113	20 (17.70)	25 (22.12)
Fenced estate	Yes	135	37 (27.41)	28 (20.74)
	No	132	23 (17.42)	24 (18.18)
Hunting season	2003–04	17	4 (23.53)	9 (52.94)
	2004–05	20	2 (10.00)	8 (40.00)
	2005–06	36	0 (0)	4 (11.11)
	2006–07	29	6 (20.69)	6 (20.69)
	2007–08	46	5 (10.87)	10 (21.74)
	2008–09	30	2 (6.67)	3 (10.00)
	2009–10	89	41 (46.07)	12 (13.48)
Age	Fawn	6	1 (16.67)	1 (16.67)
	Yearling	13	2 (15.38)	1 (7.69)
	Sub-adult	26	5 (19.23)	9 (34.62)
	Adult	222	52 (23.42)	41 (18.47)
Sex	Male	133	37 (27.82)	28 (21.05)
	Female	130	21 (16.15)	23 (17.69)
	(ND)	4	2 (50.00)	1 (25.00)
PCR	Negative	207	----	41 (19.81)
	Positive	60	----	11 (18.33)
ELISA	Negative	196	46 (23.47)	----
	Doubtful	19	3 (15.79)	----
	Positive	52	11 (21.15)	----

These results were based on the preliminary screening using the indirect enzyme-linked immunosorbent assay (ELISA) and the confirmation of results by the blocking ELISA. There was complete concordance regarding the positive results between both assays. As for the doubtful samples of the indirect test (*n* = 40), two out of the 33 from red deer were positive and 19 remained doubtful when retested by the blocking ELISA (i.e. not included in the seropositive data). The rest of doubtful samples from red deer and cattle remained negative when retested.

**Fig. 1:**
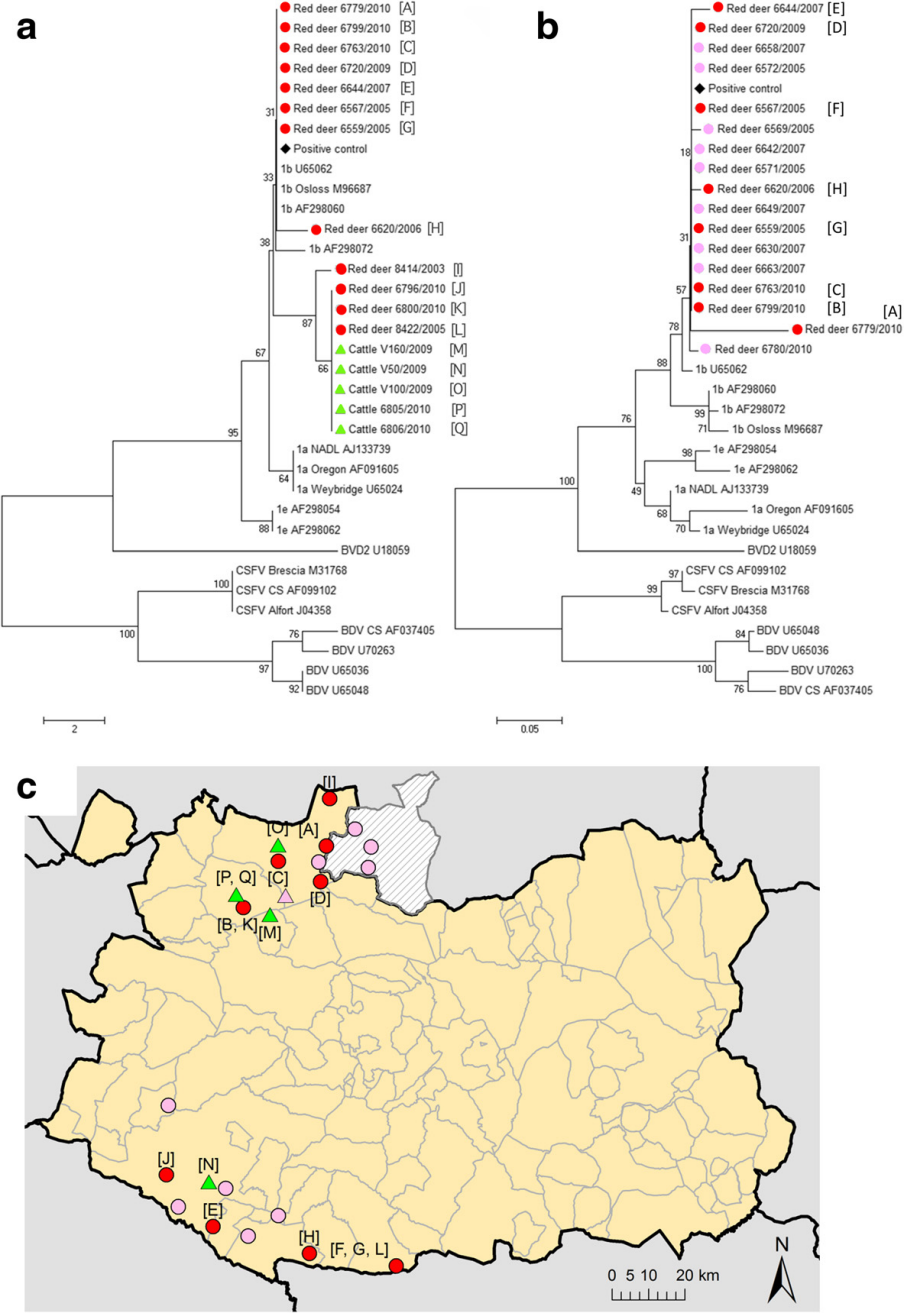
Phylogenetic trees of BVDV 5’-UTR sequences identified in red deer and cattle in the study area and pestivirus reference sequences [38]. **a** Phylogenetic tree based on sequences amplified from red deer and cattle by Taqman RT-PCR. **b** Phylogenetic tree based on sequences amplified from red deer by nested RT-PCR. Trees were constructed using the neighbor-joining algorithm [45]. Bootstrap percentage values were calculated from 1000 resamplings; values over 50 % are displayed. **c** Location of samples positive by RT-PCR that were taken from red deer and cattle. A black diamond denotes the place for the bovine field sample used as a positive control, while the grey circles indicate red deer samples, and the grey triangles samples from cattle. In the phylogenetic trees, the species, identification number and year of sampling are shown under the corresponding symbol. Letters in brackets (e.g. [A]) correspond to the sampling locations shown in the map in panel C

An approach based on the reverse transcriptase-polymerase chain reaction (RT-PCR) was used to determine the apparent prevalence of BVDV in serum samples from red deer, wild boar and cattle, as well as in tissue samples from red deer. First, samples were screened by a one-step real-time RT-PCR (rt RT-PCR). The rt RT-PCR procedure was adapted from the one described by Baxi et al. [17]. Amplicons identified as BVDV in this assay (by comparison between the melting curve of samples and controls) were then confirmed to be positive using a Taqman RT-PCR. The threshold cycle (Ct) of positive samples ranged from 33.9 to 37.1 (median, 36.4), and these values were normally higher than the ones from the positive controls. In the end, 60 red deer (22.5 %; 95 % CI = 17.5–27.5) tested positive to BVDV1 by the Taqman RT-PCR (Table 1). Fourty-three negative samples from red deer showed Ct values higher than the cutoff (37.27–39.98; median, 38.36), while the rest showed no Ct. Four out of the 17 pools of beef cattle sera as well as two samples from bullfighting bulls were positive for BVDV1 RNA detection (Fig. 1c). Results from the diagnostic tests for cattle are shown in Table 2.

**Table 2:** Diagnostic test results for BVDV detection in cattle

Variable	Category	No. samples tested by ELISA	No. ELISA-positive (%)	No. samples tested by RT-PCR^a^	No. RT-PCR-positive (%)
Production type	Bullfighting	10	0 (0)	10	2 (20)
	Beef cattle	170	82 (48.24)	17	4 (23.53)
Region	MT	140	77 (55.00)	10^b^; 13^c^	2^b^ (20); 3^c^ (23.08)
	SM	40	5 (12.50)	4^b^	1^b^ (25)
Hunting season	2008–09	80	26 (32.5)	8^c^	2^c^ (25)
	2009–10	100	56 (56)	10^b^; 9^c^	2^b^ (20); 2^c^ (22.22)

^a^Samples from the same cattle farm were pooled to perform RT-PCR because sample volume was insufficient to allow individual animal testing

^b^:Bullfighting

^c^:Beef cattle

Amplicons (68 bp) from the Taqman RT-PCR assay were sequenced for 12 red deer and five cattle. Alignment with related sequences in GenBank indicated that all samples belonged to the BVDV1b subtype. The 92.7–100 % sequence identity observed among the amplicons is consistent with the fact that the RT-PCR amplified a highly conserved region in the 5’ untranslated region (5’ UTR). Despite the short length of the sequenced region, samples were subjected to phylogenetic analysis in order to gain preliminary insights into viral molecular patterns. However, results should be interpreted with caution. All sequenced amplicons from red deer and cattle fell within subgroup 1b (Fig. 1a), which was confirmed by performing nested RT-PCR as described by Zhong et al. [18] on a set of red deer samples (Fig. 1b). However, nine of the 17 sequenced amplicons from Taqman RT-PCR clustered in a branch separate from the rest of the amplicons; these nine samples came from red deer (*n* = 4) and cattle (*n* = 5) (Fig. 1a). This grouping was not apparently related to sampling year (Fig. 1a) or location (Fig. 1c).

**Fig. 2:**
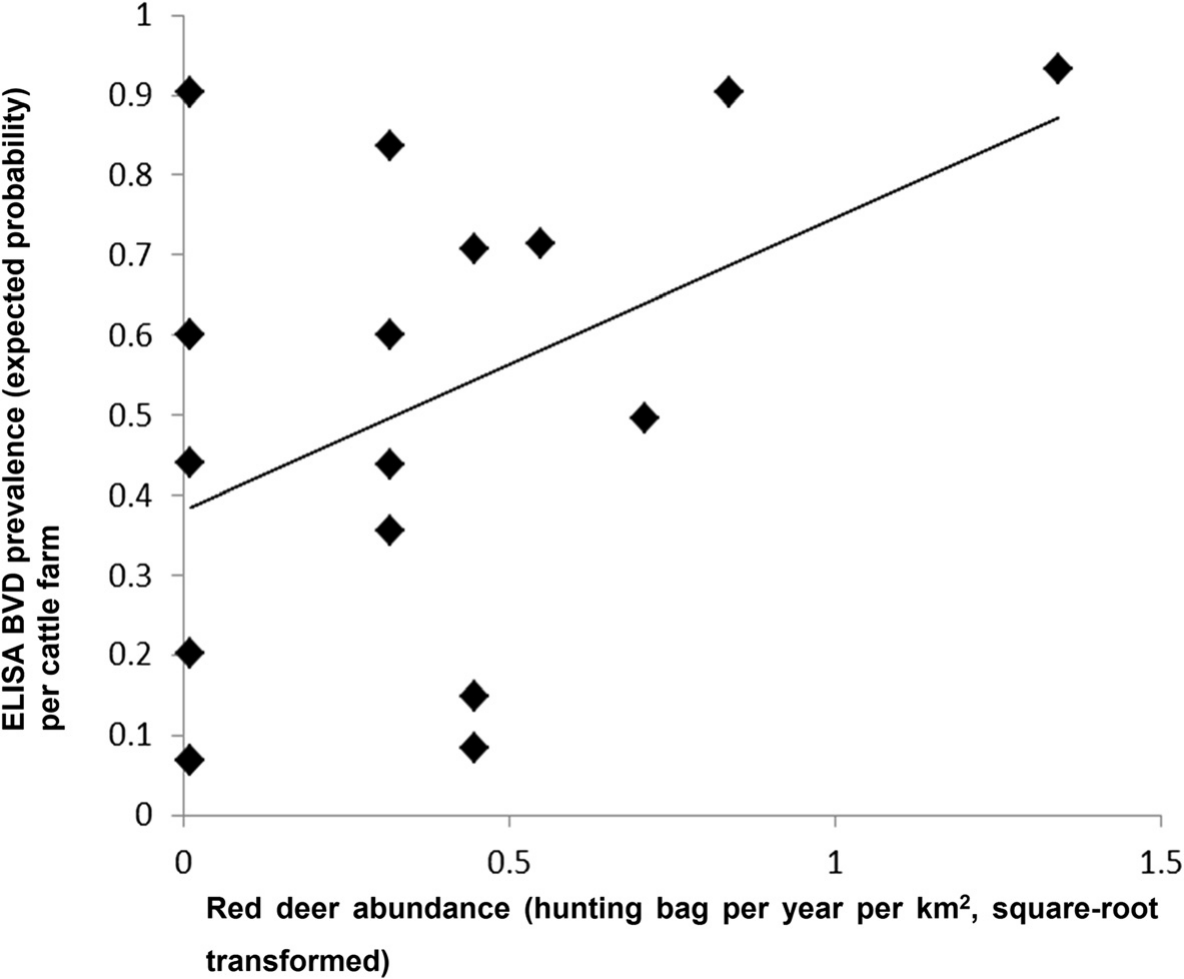
Relationship between the abundance of red deer in areas adjacent to extensive cattle farms and BVDV ELISA seroprevalence in cattle in these farms. Risk was estimated as an expected probability using a GLMM and red deer abundance data were square-root transformed

Of the 18 farms included in the study, 11 (61.1 %) recorded red deer in proximity (i.e. within 2 km). The hunting bag averaged 0.24 deer per year per km^2^ (maximum, 1.8). Abundance index of red deer showed a significant positive association with the risk that cattle would test positive for BVDV by ELISA (*F* = 6.11, d.f. = 178, *p* = 0.01, β = 28.82, 95 % CI = 6.09–51.55) (Fig. 2), although no significant relationship was found between cattle risk and red deer presence in an additional model. Female red deer showed significantly higher risk of testing positive for BVDV by ELISA or RT-PCR than did males (*F* = 5.45, d.f. = 258, *p* = 0.02, β = −0–77, 95 % CI − 1.43 to − 0.13; males served as the reference).

## Discussion

While the role of cattle in BVDV maintenance is well established, the epidemiology of wild ruminants is not so clear and more difficult to assess. In this study, we determined seroprevalence and apparent prevalence of BVDV in populations of extensively raised cattle, red deer and wild boar living close to one another in south-central Spain. Our results suggest that BVDV is actively circulating in this area in both domestic and wild ruminants. In particular, BVDV infection in red deer seems endemic throughout the area, affecting more than 22 % animals regardless of age. Preliminary phylogenetic analysis based on 68-bp viral sequences suggests that the BVDV strains circulating among red deer and cattle may be related, though more extensive sequencing studies are required to verify this. The abundance of red deer living in proximity to extensively raised cattle significantly affects the likelihood that those cattle will test positive for BVDV. These findings suggest that wildlife-livestock interactions may contribute to the epidemiology of BVD in south-central Spain. This further supports the potential usefulness of BVDV as a health marker to monitor interactions between cattle and wildlife such as red deer.

Seroprevalence of BVDV in our study was 45.6 % in cattle and 19.5 % in red deer. This is high relative to the prevalence of anti-pestivirus antibodies, including against BVDV, reported in deer populations in many other parts of Europe [8, 19, 20]. However, several other studies have detected seroprevalences up to 60 % in free-ranging wild ruminant populations [11], including deer species [21]. These high prevalences may indicate endemic BVDV circulation. Previous results in the literature are based mostly on ELISA, which can show false positives due to cross-reactions against other pestiviruses including BDV [22]. Therefore we confirmed our positive ELISA results in the present study using a blocking kit, which is highly recommended for its use in wildlife [8], and there was complete concordance of results.

Consistent with high BVDV seroprevalence in deer, these animals also showed 22.5 % apparent prevalence, which we consider to be reliable, given that it is based on a Taqman probe and subsequent sequencing of the PCR products. Taking both values together (seroprevalence and apparent prevalence), endemicity of BVDV in the study area is suggested. Ct values in positive animals were relatively high (# 33.9), suggesting that viral load (or the amount of RNA) found in the samples was low. Such high values may reflect non-specific amplification reactions, but this seems unlikely as the assay has been carefully optimized to reliably detect low number of viral RNA copies [17].

We did not find significant age variation in prevalence among red deer. Other studies showed that infected deer may survive until adolescence [21, 23–26]. Nonetheless, our sampling size for fawns was limited, so future research should address this age variability in detail. Apart from that, high prevalences were maintained from yearlings to adults, evidencing that infection occurs at early ages. However, prevalence in red deer showed a significant bias toward females. This sex difference may reflect, in part, differences in herd structure and behavior: female deer tend to reside at relatively high densities in larger herds, which may enhance viral circulation [27–29].

A substantial proportion of red deer [*n* = 41 (15.4 %)] was found to be positive only by the ELISA. These infections may have occurred long before the study, since anti-BVDV antibody titers can persist for years, at least in cattle [3]. In addition, a small number of red deer [*n* = 11 (4.1 %)] tested positive by both ELISA and RT-PCR; these animals may have been sampled during the last phase of viremia, when antibodies production was just starting. This finding would be likely in the case that viremia lasts longer in red deer than in cattle (which lasts between four and 15 days after the acute infection [30, 31]). Another explanation for why animals tested positive by both ELISA and RT-PCR may be that they were PI fawns that had been infected only a few weeks before. This possibility seems less likely, however, since all 11 animals were older than 6 months, and colostral antibodies in deer last as long as in cattle [32]. A third potential explanation may be related to previously BVDV-exposed individuals recently infected by a different strain.

A substantial proportion of red deer (i.e. *n* = 46, 17.2 %) tested positive only by RT-PCR, showing no detectable antibody titers. This may reflect infections still in their first week, when antibodies were not circulating yet, or it may reflect persistent infection. The first possibility is not very likely given the low probability of capturing such a large proportion of infections within the first week. The second one would imply that PI deer could survive to adulthood, considering that 41 out of these 46 animals were older than 2 years. These results highlight the need of more studies on immunity and pathogenesis of BVDV infections in deer, including persistence of viremia.

The combination of these diagnosis results and high Ct values suggest that the infections detected in the present study reflect viral circulation for at least 8 years rather than a recent outbreak of acute infections. Extended viremias might be responsible for high apparent prevalences in cattle, related to the BVDV long-term persistence in bovine white blood cells [33]. Nevertheless, these findings should be studied and verified in red deer and extended by further serological and molecular analyses, especially viral isolation, in order to clarify BVD epidemiology in the region. Preferably these future studies should be longitudinal and should examine viral infection in greater detail. The insights may help prevent BVDV spread via red deer.

Our failure to detect BVDV in wild boar by either ELISA or RT-PCR is consistent with BVDV seroprevalences of 0.6–4.5 % in the Czech Republic [34], Croatia [35] or Germany [36]. Given that our wild boar samples were collected near hunting estates where BVDV was circulating in red deer, we conclude that wild boar is unlikely to act as a BVDV reservoir. However, attention should be paid to the cross reactions against CSFV [37].

Though virus isolation has not been performed in this study, and amplicon sequences were short, the phylogenetic analysis suggested that all the samples studied belonged to subgroup 1b. However, several samples from cattle and red deer were grouped together in a branch of the phylogenetic tree (Fig. 1a). This is consistent with the idea that a BVDV strain was circulating within the cattle and red deer populations, but this must be verified through more extensive sequencing. Viruses belonging to this subgroup 1b are widespread in cattle over Europe, including Spain [38, 39]. However, it seems that a different BVDV type could be circulating only among red deer. In the case of roe deer, studies in Germany [23], Norway [40] and Switzerland [20] indicate that the infection in this wild host can be caused by wild BVD-like strains. Further studies could focus on obtaining longer sequences and more accessible information on pestiviral strains, not only considering the 5’ UTR, but also other genes, such as the N-terminal autoprotease [41]. These studies are needed in order to fully understand the patterns of BVDV circulation and to see whether there is a different pathogen circulating among different wild species and to assess their potential impact in domestic livestock.

Of the 18 farms in our study, 11 (61.1 %) recorded the presence of red deer within a radius of 2 km. Indeed, in south-central Spain, deer populations often reside in and near farms with extensively raised cattle [42]. We observed that the abundance index of red deer showed a significant positive association with the likelihood that cattle would test positive for BVDV. While many factors are likely to influence the number of deer hunted per year and the hunting bag data are therefore likely to provide only a crude indicator of red deer abundance, the positive relationship between deer hunting bag and cattle BVD seroprevalence suggests that the presence of deer may affect the likelihood of BVD infection in cattle. This fact supports the molecular findings suggesting that BVDV transmission occurs at the wildlife-livestock interface. If PI individuals were common among red deer, as our immunological results suggest, it could highlight the role of red deer as reservoir for BVDV. Indeed, red deer may be a true reservoir in our study area, given that they are endemic to the area, and the risk that red deer would test positive for BVDV did not correlate with abundance of cattle or other domestic ruminants in the hunting estates.

Nonetheless, in this correlational study, we cannot infer whether red deer are responsible for transmitting BVDV to cattle. However, in the light of our results, these free-living wild hosts could act as a hidden reservoir of BVDV for domestic animals, as others have previously pointed out [7, 8, 43, 44]. A role for red deer as BVDV reservoir seems more likely in certain epidemiological scenarios, like that in our study area, where both wild and domestic ruminants share a habitat under specific management practices, which is ideal for the virus circulation and maintenance.

## Conclusions

In this study we used molecular microbiology, immunology and field epidemiology methods to assess BVDV as a potential marker for monitoring the contact between wildlife and cattle in south-central Spain. Our results suggest that BVDV is infecting red deer populations, and some strains could be circulating among the wildlife. The possibility that the same BVDV strain infected the cattle and red deer in our study area highlights the relevance of red deer as a potential source of infection to cattle. Interactions between extensively raised cattle and wildlife may contribute to BVDV spill back, with implications for BVD surveillance and control programs, as well as wildlife conservation plans. More research is needed to understand how wildlife management, farming practices, habitat, and species-specific behavior and interactions influence BVDV infection in wildlife and cattle.

## Methods

### Study area and animal sampling

**Fig. 3:**
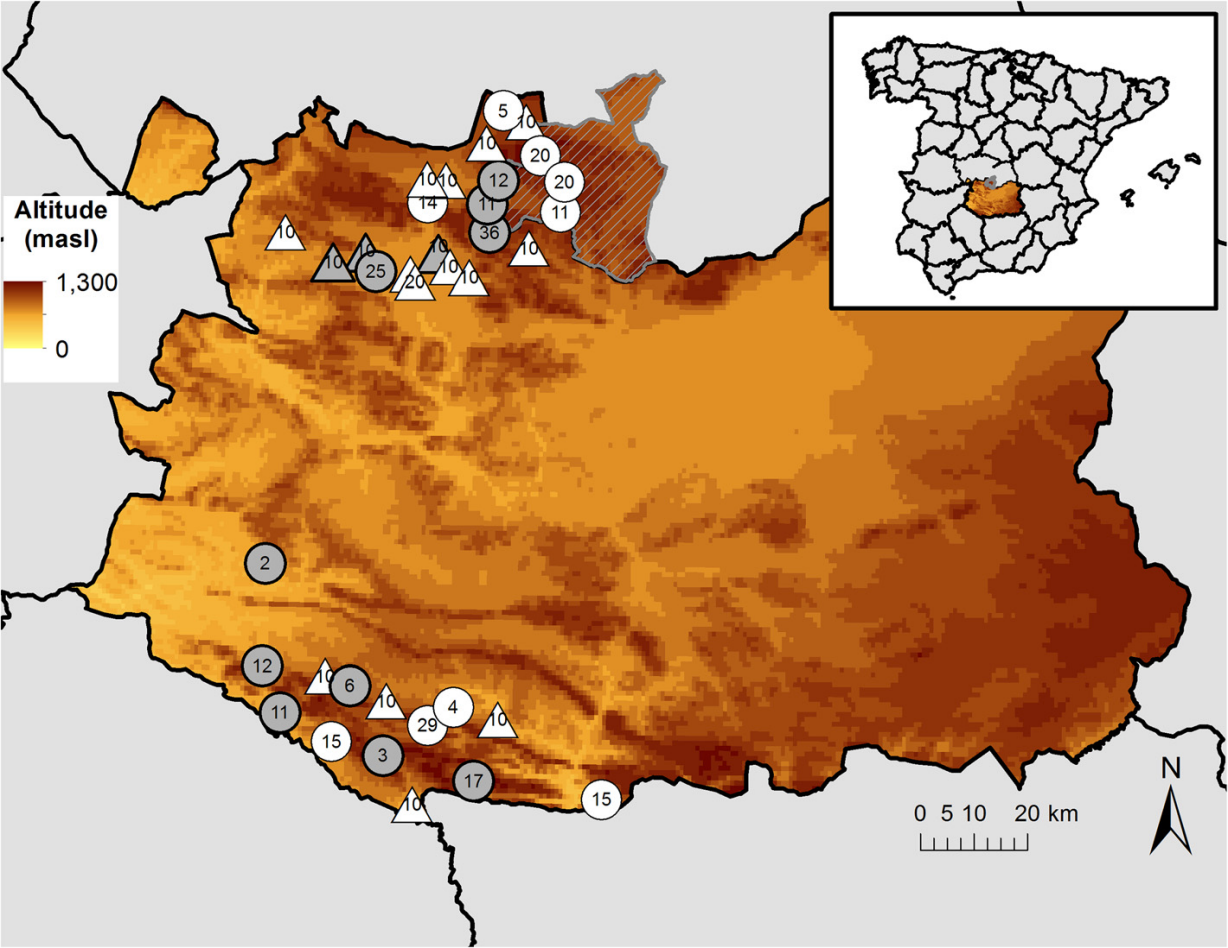
Location of sampling sites in Castile-La Mancha. Most samples were collected in two of the highest areas of the region: Montes de Toledo, entailing the northern part of Ciudad Real and the Toledo municipality of Los Yébenes (grey hatched), and Sierra Morena in southern Ciudad Real. Circles denote hunting estates, and triangles denote cattle farms. The numbers inside each figure indicate the number of sampled red deer and cattle per hunting estate or farm, respectively. Figures in white indicate hunting estates lacking fence enclosures; black-outlined figures in grey, hunting estates with fence enclosures. The inset in the upper right shows the location of the study area in south-central Spain

The study was carried out in the provinces of Toledo (TO) and Ciudad Real (CR) in the Castile-La Mancha region (CLM) (38° 32’ 45” N to 39° 32’ 45” N in latitude; 3° 54’ 34” W to 4° 52’ 18” W in longitude) in south-central Spain (Fig. 3). The study area is flanked by two mountain chains: Montes de Toledo (MT), located between TO and northern CR; and Sierra Morena (SM), in the southern part of CR (Fig. 3), which comprises interspersed hunting estates and farms.

Hunted red deer (*n* = 267) and wild boar (*n* = 52) were sampled from 2003 to 2010 in 19 hunting estates from CLM, comprising 9 estates in MT and ten in SM. We considered hunting an adequate method for surveying wild ungulate populations given that animals were shot randomly during hunting drives. Hunting took place from October to February. Red deer and wild boar were necropsied in detail and sex was recorded for all animals except one red deer and three wild boar. Based on tooth eruption patterns [45], red deer were classified as fawns if younger than 1 year (*n* = 6), as yearlings if between 1 and 2 years (*n* = 13), as sub-adults if between 2 and 3 years (*n* = 26), and as adults if over 3 years (*n* = 222). Wild boar were classified as piglets if younger than 6 months (*n* = 1), as weaners if between 7 and 12 months (*n* = 11), as juveniles if between 12 and 24 months (*n* = 15), and as adults if over 2 years (*n* = 22).

Blood samples were taken from the heart or thoracic cavity during field necropsies, and sera were stored at −20 °C for further analysis. In several red deer, tissue samples were also included, i.e. spleen (*n* = 6) and retropharyngeal lymph nodes (*n* = 10), and conserved at − 20 °C until the homogenization in sterile PBS in a 1:10 proportion.

Serum samples from 18 extensive cattle farms were obtained as part of regular health measures to monitor bovine tuberculosis. Ten samples from each farm were obtained and stored at − 20 °C until analysis. Since the volume of collected serum proved insufficient to allow virological analysis of individual animals, samples from all animals within one farm were pooled for that part of the study (see below).

### Serological analysis

Serological analyses to detect the presence of antibodies against BVDV were performed in all animals, including red deer (*n* = 267), wild boar (*n* = 52) and cattle (*n* = 180), using an indirect ELISA (IDEXX, Liebefeld-Bern, Switzerland). This kit detects antibodies targeted against BVDV-1 and − 2 strains. A blocking ELISA (INGENASA, Madrid, Spain), which detects specific antibodies against the p80 antigen of the BVDV, was also performed in order to clarify doubtful results (seven cattle and 33 red deer), as well as confirm samples that were positive in the indirect ELISA (82 cattle and 50 red deer) . Both kits were used according to the manufacturers’ instructions. Manufacturer-specified sensitivity and specificity were 96.3 and 95 % for the indirect ELISA, and 99.5 and 92 % for the blocking ELISA.

### Virological analysis

Prior to the virus analyses, total RNA was extracted from serum samples using the Nucleospin® RNA virus kit (Macherey-Nagel, Düren, Germany) and from tissue homogenates using the Nucleospin® RNA II kit (Macherey-Nagel). We followed the manufacturer’s recommendations to perform the extractions. Extraction products were stored at − 20 °C until further analysis.

Samples were first screened for the presence of BVDV using an rt RT-PCR assay, which was developed by adapting the RT-PCR described by Baxi et al. [17]. Our rt RT-PCR amplified a 68-bp product (106 bp including primer-derived sequences) from the 5’ UTR, which is conserved among all pestiviruses, and then detected the amplicon using SYBR Green. This technique was used for a preliminary screening of samples. The optimized rt RT-PCR reaction (25 μl) contained 2 μl of RNA template, 12.5 μl of SYBR Green Master mix (Quantitative RT-PCR Brilliant II SYBR Green Master Mix), 0.0625 μl of StrataScript RT/RNase Block Enzyme Mixture (both from Stratagene, Palo Alto, CA, USA) and 400 nM of each primer. Reactions were performed in a Stratagene MxPro 3000 thermocycler (Stratagene, Palo Alto, CA, USA) using the primers and probe in Table 3. Thermal cycling conditions were as follows: 30 min at 48 °C for the RT, followed by 10 min at 95 °C for denaturalization; then 40 amplification cycles of 95 °C for 30 s, 60 °C for 60 s, and 72 °C for 30 s; and a final extension at 72 °C for 7 min. Fluorescence was measured at the extension step of each cycle. Dissociation curves were generated by increasing the temperature from 65 °C to 99 °C over 35 cycles, with 1 °C increments every 30 s.

**Table 3:** Primer and probe sequences for RT-PCR-based detection of BVDV

ID	Sequence (5’ - 3’)	Size (bp)	Amplicon size (bp)	Region amplified	Target viruses	Source
Pesti-FW	5’-CTAGCCATGCCCTTAGTAG-3’	19	106	5’-UTR	Common for Pestivirus	[17]
Pesti-RS	5’-CGTCGAACCAGTGACGACT-3’	19				
FAM-BVDV1	5’-FAM-TAGCAACAGTGGTGAGTTCGTTGGATGGCT-BHQ-3’	30			BVDV-1	

After samples were screened using rt RT-PCR, they were confirmed to be BVDV1-positive using a Taqman-based RT-PCR assay [17]. Samples whose dissociation curves were similar to the ones produced by the positive controls were especially considered for confirmation, although other samples were also included in order to check the good performance of the assays. The Taqman-based RT-PCR assay has been shown to correlate perfectly with the results of virus isolation [17]. We tested for BVDV1, since it accounts for 90 % of the BVDV isolates in Europe [8]. The final Taqman RT-PCR reaction (20 μl) contained 2 μl of RNA template, 10 μl of Kapa Probe Fast PCR Master Mix (Kapa Biosystems, Woburn, MA, USA), RT Kapa SYBR Fast One-Step qRT-PCR (Kapa Biosystems), 200 nM of each primer and the BVDV1-specific probe (Table 3). Reactions were performed in a Stratagene MxPro 3000 thermocycler. Thermal cycling conditions were as follows: 15 min at 42 °C for RT, 5 min at 95 °C for denaturalization; then 40 amplification cycles of 95 °C for 3 s and 60 °C for 30 s. The detection limit of the Taqman assay was determined to be 100 tissue culture infectious dose 50 (TCID_50_), corresponding to a Ct value of 37.72 ± 0.58 [17]. We considered a sample positive when its Ct in the Taqman-based RT-PCR assay was lower than 37.1.

Controls were included from the RNA extraction through the RT-PCR to rule out contamination. As positive controls, we used two bovine field-isolated strains belonging to the BVDV1b as positive controls. As negative controls, we used sera from a bottlenose dolphin (*Tursiops truncatus*). No-template controls were included in every step and were placed in between the samples and the controls, to monitor the good management during the performance of the techniques. The controls had to perform as expected in order for the RT-PCRs to be accepted as valid.

Taqman RT-PCR products from 17 samples were confirmed by electrophoresis, using 10 μl of the RT-PCR reaction in a 3 % agarose gel (with an expected amplicon size of 106 bp), and subsequently gel-purified and sequenced.

### Phylogenetic analysis

A phylogenetic tree was calculated using the sequences of the abovementioned 17 positive samples (12 from red deer and five from cattle). Since these amplicons were not long enough to create a definitive tree, we also sequenced 18 positive samples from red deer using a nested RT-PCR [18]. This assay produced an amplicon of 195 bp (159 bp excluding the primer-derived sequences) from 5’ UTR.

Both sets of sequences were aligned with DNA sequences from the GenBank database using Clustal X, version 1.83. Several pestivirus reference and field sequences for BVDV1 (subgroups a, b, and e), BVDV2, CSFV and BDV were used in the alignment files [39]. Phylogenetic analyses were performed using the Kimura model of nucleotide substitution, and phylogenetic trees were constructed using the neighbor-joining method in MEGA5 software [46]. The statistical significance of the tree topology was evaluated by bootstrap re-sampling of the sequences 1000 times. The cutoff value was 50 %. The results and sequences are shown in Fig. 1.

### Statistical analysis

For each farm, we collected data about location (x and y coordinates of the farm centroid) and abundance of wildlife species in nearby hunting estates (i.e. within a radius of 2 km). This radius was chosen based on the spatial distribution of red deer in extensive farms in the study area (the authors, unpublished). We calculated an index of relative abundance for red deer per study farm by calculating the overall annual hunting bag (deer hunted per year per km^2^) from the nearby hunting estates during the study period. Details on converting hunting bags into an abundance index have been presented elsewhere [47]. We used a GIS layer including the perimeters of all hunting estates, which are the smallest management units. Data from hunting estates were processed using ArcGIS 9.2 (ESRI, Redlands, CA, USA).

We used a generalized linear mixed model (GLMM) to explore a possible relationship between red deer abundance near a farm (square-root transformed, explanatory variable) and the risk that cattle on that farm would test positive for BVDV by ELISA (binomial response variable). Farm and sampling season were included as random factors. Similar models were created to identify factors affecting the risk that individual red deer would test positive for BVDV by ELISA or RT-PCR. Factors included sex, age, deer abundance index and livestock density in the estate. We used hunting estate and season as random factors.

We used binomial error and a logit link in all models. SPSS 19 (Surrey, UK) was used to fit the models. All reported p values were two-sided, and p values < 0.05 were considered statistically significant.

## Abbreviations

5’ UTR: 5’ untranslated region
bp: Base pair
BDV: Border disease virus
BVDV: Bovine viral diarrhea virus
CI: Confidence interval
CLM: Castile-La Mancha
CR: Ciudad real
CSFV: Classical swine fever virus
Ct: Threshold cycle
ELISA: Enzyme-linked immunosorbent assay
GIS: Geographic information system
GLMM: Generalized linear mixed model
MT: Montes de Toledo
PI: Persistently infected
RNA: Ribonucleic acid
rt RT-PCR: One-step real-time reverse transcriptase-polymerase chain reaction
SM: Sierra Morena
TCID_50_: Tissue culture infectious dose 50
TO: Toledo
